# Developing and adopting safe and effective digital biomarkers to improve patient outcomes

**DOI:** 10.1038/s41746-019-0090-4

**Published:** 2019-03-11

**Authors:** Andrea Coravos, Sean Khozin, Kenneth D. Mandl

**Affiliations:** 1 Computational Health Informatics Program, Boston Children’s Hospital, Boston, MA USA; 2 Harvard-MIT Center for Regulatory Science, Boston, MA USA; 3 Food and Drug Administration, Silver Spring, MD USA; 4 Department of Biomedical Informatics, Harvard Medical School, Boston, MA USA

**Keywords:** Diagnostic markers, Policy

## Abstract

Biomarkers are physiologic, pathologic, or anatomic characteristics that are objectively measured and evaluated as an indicator of normal biologic processes, pathologic processes, or biological responses to therapeutic interventions. Recent advances in the development of mobile digitally connected technologies have led to the emergence of a new class of biomarkers measured across multiple layers of hardware and software. Quantified in ones and zeros, these “digital” biomarkers can support continuous measurements outside the physical confines of the clinical environment. The modular software–hardware combination of these products has created new opportunities for patient care and biomedical research, enabling remote monitoring and decentralized clinical trial designs. However, a systematic approach to assessing the quality and utility of digital biomarkers to ensure an appropriate balance between their safety and effectiveness is needed. This paper outlines key considerations for the development and evaluation of digital biomarkers, examining their role in clinical research and routine patient care.

## Introduction

Biomarkers are characteristics (such as a physiologic, pathologic, or anatomic characteristic or measurement) that are objectively measured and evaluated as an indicator of normal biologic processes, pathologic processes, or biological responses to a therapeutic intervention.^1^ Building on this standard definition, we describe an emerging class of biomarker, the “digital biomarker”, which has important implications for both clinical trials and clinical care. “Digital” refers to the method of collection as using sensors and computational tools, generally across multiple layers of hardware and software. The measurements are often made outside the physical confines of the clinical environment using home-based connected products^2^ including wearable, implantable, and ingestible devices and sensors. Digital biomarkers span a broad range of diagnostic and prognostic measurements (Table 1). We discuss development and evaluation of the digital biomarkers, outlining opportunities and challenges associated with their use in clinical research and routine care. As remote monitoring of digital biomarkers becomes increasingly prevalent, we discuss the challenges to patient privacy and patient autonomy.

**Table 1:** Digital biomarker examples

Category^a^	Definition^a^	Example^a^	Corresponding Digital Biomarker Examples
**Susceptibility/Risk Biomarker**	A biomarker that indicates the potential for developing a disease or medical condition in an individual who does not currently have clinically apparent disease or the medical condition.	Breast Cancer genes 1 and 2 (BRCA1/2) mutations may be used as a susceptibility/risk biomarker to identify individuals with a predisposition to develop breast cancer.	[*] Detect cognitive changes in healthy subjects at risk of developing Alzheimer's disease using a video game platform.^18^
			[**] Classify adults at high risk of late-onset Alzheimer's disease using computerized cognitive testing.^19^
			[*] Reduce key risk metrics for anterior cruciate ligament injury during jump landings using inertial sensor-based feedback.^20^
**Diagnostic Biomarker**	A biomarker used to detect or confirm the presence of a disease or condition of interest or to identify individuals with a subtype of the disease.	Repeated blood pressure readings obtained outside the clinical setting in adults 18 years and older may be used as a diagnostic biomarker to identify those with essential hypertension.	[*] Diagnose ADHD in children using eye vergence metrics.^21^
			[*] Detect arrhythmias using convolutional neural networks and a wearable single-lead heart monitor.^22^
			[*] Detect depression and Parkinson’s disease using vocal biomarkers.^23^
			[*] Diagnose asthma and respiratory infections using smartphone-recorded cough sounds.^24^
**Monitoring Biomarker**	A biomarker measured serially for assessing the status of a disease or medical condition or for evidence of exposure to (or effect of) a medical product or an environmental agent.	Prostate-specific antigen (PSA) may be used as a monitoring biomarker when assessing disease status or burden in patients with prostate cancer.	[**] Monitor signs of Parkinson's disease using smartphone-based measurements.^25^
			[*] Quantify Parkinson’s disease severity using smartphones and machine learning.^3^
			[**] Track time and location of short-acting beta-agonist inhaler use using an attached wireless sensor.^26^
			[*] Predicting sleep/wake patterns from a 3-axis home-based accelerometer using deep learning.^27^
			[*] Detection of nocturnal scratching movements in patients with atopic dermatitis using accelerometers and recurrent neural networks.^28^
**Prognostic Biomarker**	A biomarker used to identify the likelihood of a clinical event, disease recurrence, or progression in patients who have the disease or medical condition of interest.	Increasing prostate-specific antigen (PSA) may be used as a prognostic biomarker when evaluating patients with prostate cancer during follow-up, to assess the likelihood of cancer progression.	Stratify mental health conditions and predict remission using passively collected smartphone data.^29^
			Detect post-acute care deterioration in patients at home, applying machine learning to multi-sensor digital ambulatory monitoring.^30^
**Predictive Biomarker**	A biomarker used to identify individuals who are more likely than similar individuals without the biomarker to experience a favorable or unfavorable effect from exposure to a medical product or an environmental agent.	Human leukocyte antigen allele (HLA)–B*5701 genotype may be used as a predictive biomarker to evaluate human immunodeficiency virus (HIV) patients before abacavir treatment, to identify patients at risk for severe skin reactions.	Predict autism risk in the siblings of children with autism, using an EEG biomarker.^31^
			Detect asymptomatic atrial fibrillation (AF) as a stroke risk factor, remotely through a connected device.^32^
**Pharmaco-dynamic/Response Biomarker**	A biomarker used to show that a biological response has occurred in an individual who has been exposed to a medical product or an environmental agent.	Blood pressure may be used as a pharmacodynamic/response biomarker when evaluating patients with hypertension, to assess response to an antihypertensive agent or sodium restriction.	Measure cognitive performance with the Cambridge Neuropsychological Test Automated Battery (CANTAB) to test the effects of erythropoietin.^33^
			Measure blood pressure using a digital sphygmomanometer to assess response to antihypertensive therapy.^34^

^a^Selected from the FDA-NIH “Biomarkers, EndpointS, and other Tools” (BEST) classification for traditional biomarkers

[*] Digital biomarker under development

[**] Digital biomarker in use (in a clinical trial or an FDA cleared/approved digital health product, or a digital health app in use not requiring approval)

Just as clinicians must evaluate a drug’s safety and effectiveness by critically appraising clinical trials, they will increasingly need to know how to evaluate, select, and “prescribe” digital health tools and biomarkers. Some biomarkers are immediately familiar to patients or physicians as they are digitized versions of well-established metrics—for example, glucometer readings transmitted by Bluetooth, or the timed six-minute walk test measured with the smartphone’s built-in gyroscope and accelerometer. Others, such as the smartphone-derived tapping test for Parkinson’s disease severity, are novel and evolving.^3^ Digital biomarkers are an essential component in autoregulated closed loop systems. For example, in an “artificial pancreas” model, a continuous glucose sensor linked to an insulin pump can automatically dose insulin in patients with diabetes.^4^

## The anatomy and evaluation of digital biomarkers

### Measurements

An input layer such as a camera, microphone, or sensor captures a digital biomarker signal. For example, photoplethysmographs measure blood volume changes in the microvasculature using an optical sensor placed on the skin surface. A signal processing layer, typically an algorithm, converts the input signal into actionable metrics (e.g., oxygen saturation and/or heart rate), or digital biomarkers. Although measuring blood volume changes using photoplethysmography is widely accepted in medical practice, the interplay among hardware, sensors, and algorithms can make the evaluation of emerging digital biomarkers difficult. There are several challenges in deciding not only whether a digital biomarker is valid, but equally important, whether it is “fit-for-purpose”, meaning that the product has an explicit context of use, meets appropriate requirements for accuracy and precision, and is accompanied by the metadata needed for analysis and interpretation.^5^

### Verification

Analytical verification uses engineering bench tests to ensure that the product is measuring and storing values accurately by confirming the tool’s accuracy, precision, and reliability. Confidence in the performance of digital biomarkers is an important consideration for researchers, clinicians, and patients. For example, the verification step ensures that the translation from raw data, e.g., that a heart rate sensor measuring electrical potential in millivolts, faithfully converts that signal into an accurate heart rate, expressed in beats per unit of time.

### Validation

As with diagnostics, the performance of digital biomarker algorithms may vary across different patient populations, producing different rates of false-positive or false-negative outputs in different groups. Validation addresses whether the measurement is applicable in the target population and context of use,^6^ which would render digital biomarker “fit for purpose”. For example, a tool measuring sleep and waking periods perform against polysomnography may perform differently in a patient population with insomnia versus sleep apnea versus healthy volunteers.

### Modularity

Digital biomarker products can be composed of multiple individual software and hardware components. When the components are interoperable, they can be mixed and matched as modular components to assemble a diverse array of offerings. For example, the US Food and Drug Administration (FDA) recently approved the Dexcom integrated continuous glucose monitoring system as the first type of continuous glucose monitoring system that can be used in a modular fashion with other compatible medical devices and electronic interfaces, including automated insulin dosing systems and diabetes management devices.^7^

**Fig. 1:**
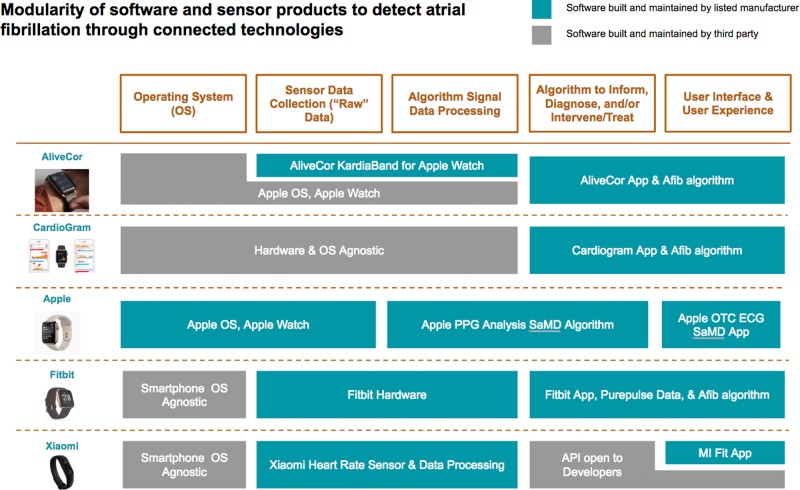
Digital biomarker products. Five products, all detecting a similar digital endpoint, are constructed with differing, modular approaches. In the first column are five products to detect atrial fibrillation: AliveCor, CardioGram, Apple Watch plus ECG App, Fitbit, and Xiaomi. Across the top, are major software modules comprising the product, from the operating system on the left to the user interface on the right. Some modules are created by the product manufacturer and others by a third party. If the listed organization manufacturers the component, the module is represented in green. If instead it is created by a different party, the color is gray. These differently composed products require different strategies for verification, validation, and likely also regulatory clearance. Figures are reused with permission from the copyright owners, and the Apple watch image is Courtesy of Apple Inc

Software and hardware manufacturers have started to specialize in modular pieces of a connected product’s data flow tool chain (Fig. 1).

### Regulation of modular components

The FDA regulatory process can often address particular, modular, components along a digital biomarker’s measurement apparatus. The FDA is piloting a program that would “pre-certify” companies and their policies^8^ in order to offer a streamlined path to market for their product-level approvals and modifications.

Historically, most of the software-products have been categorized as software in a medical device (SiMD), which operates the device and sensors (e.g., firmware). More recently, digital biomarker components are categorized as software as a medical device (SaMD) solutions. SaMDs can perform a medical function without being part of a hardware medical device (e.g., machine-learning based tools in mobile apps^8^) have novel properties and potential for wider adoption. Definitions distinguishing SaMD from SiMD are evolving. The FDA recently cleared two SaMDs compatible with the Apple Watch for detection of atrial fibrillation. The first is an “over the counter” electrocardiogram app for display of atrial fibrillation^9^ and the second can notify the user of an irregular rhythm.^10^ The hardware, the Apple Watch, serves as a component supporting digital biomarker measurement. The Apple Watch over the counter EKG app and irregular rhythm notifications a re FDA cleared as SaMDs.

While modularity enables mixing and matching across a variety of components, it can also be a source of potential error. For example, performance changes to an operating system may affect the speed of computation^11^ and, for example, corrupt measurement of a Parkinson’s tapping test, which uses a smartphone to calculate a digital biomarker based on timed reaction.

## Potential benefits and risks of digital biomarkers

As new modalities are incorporated into connected devices, mobile apps, and software products for patients at home, a natural area of growth in biomarker collection is remote collection of patient-generated measurements. As digital biomarkers are increasingly used as endpoints in clinical trials, we anticipate that clinicians will have a growing number of validated means of gathering clinical insights on patients remotely. However, incorporation of these tools in clinical research is dependent on accelerating the development of new study designs such as those employed in decentralized clinical trials, where many of the trial participant touchpoints occur at home.^12^ Furthermore, verification and validation of digital biomarkers require a uniquely collaborative approach, with engineering, data science, health information technology, and clinical research functions tightly coordinated as integrated multidisciplinary units.

New digital biomarkers are directly targeting clinical management. The Empatica Embrace Watch, for example, is a “smartband” wrist-device that measures sympathetic nervous impulses at the skin and infers parasympathetic activity from heart rate variation. Its algorithm detects seizures and its associated app suite can alert care providers. There are many examples of digital biomarkers in use or actively under development today, as well as computational metrics with potential for development into digital biomarkers (Table 1). We expect that as digital biomarkers become increasingly used in clinical trials, patient and physician adoption will increase in care and self-management. Digital tools also allow deep collection of data on individual trial participants as well as patients in clinical settings, thereby providing an opportunity for “N of 1” clinical investigations, the cornerstone of evidence generation for personalization of care.

As new platforms for connected technologies emerge, “composite” biomarkers simultaneously incorporating multi-sourced physiologic parameters (e.g., blood pressure, heart rate, and oxygen saturation) and patient-reported information can have higher diagnostic and prognostic value. With more data, an algorithm’s accuracy improves. For example, incorporation of the user’s height, weight, age, and gender increases step count accuracy, because a 25-year-old’s gait is not equivalent to that of an 80-year-old. Availability of contextual information will enable more personalized algorithms (e.g., a step count algorithm designed for a population with late-stage Parkinson’s), and also can combine data sources to create novel measures for conditions that have historically struggled to have meaningful endpoints (e.g., brain and nervous system disorders).

Ensuring privacy and autonomy is paramount as digital biomarkers are incorporated into care and self-management, and incentive programs encouraging wellness and treatment plan adherence. While healthcare delivery organizations using digital biomarkers are of course Health Insurance Portability and Accountability Act (HIPAA) covered entities, when citizens engage directly with the technologies or technology companies, HIPAA does not apply.^13^ Social media and targeted advertising platforms typically employ end-user-license agreements and terms of service to outline data-sharing rights and privacy policies. However, like informed consent, health data rights should cover a continuum of activities over time. Therefore, data use agreements for digital biomarker development should contain clear statements on conditions for data usage especially for tools that collect near-continuous data, like movement, voice, and other sensitive biometric states.

Connected software products may pose cybersecurity challenges exposing trial participants and patients to privacy breaches or even safety risks. Just as HIPAA and the Common Rule are written to protect a patient’s medical record data and biospecimens, nascent efforts are building protections for digital “specimens”. New frameworks are emerging around the security,^14^ ethics,^13^ and informed consent challenges,^15^ of digital phenotyping technologies.^16^ One approach—a promising one for tracking security vulnerabilities and issues of performance, transparency, and accuracy—would require software manufacturers to provide, in premarket submission to the FDA, a “Software Bill of Materials” which is analogous to the ingredient list for a medication.^17^

A challenge to the evaluation of algorithms is that many are proprietary, patented or are trade secrets. For example, the AliveCor, Cardiogram, and Apple atrial detection algorithms and training data sets, for example, are not published. Instead, these companies offer a textual description of what the code does. The Empatica epilepsy monitor, for example, does not readily output raw signal, but instead, only the processed output interpreted by its proprietary algorithm. Hence the impact on a population of a digital biomarker-driven clinical management plan may not always be transparent to patients and clinicians. Testing characteristics, including selected thresholds for action, sensitivity, and specificity should be made transparent to the healthcare professional, regulators, and trial participant and patient users of digital biomarkers.

## Conclusion

In recent years, digital biomarker development has begun integration into translational and clinical research. An increasing number of industry and academic investigators are at the leading edge of a new wave of innovations.

To accrue maximum benefit to the patient, a safe and effective digital biomarker ecosystem requires transparency of the algorithms, interoperable components with open interfaces to accelerate the development of new multicomponent systems, high integrity measurement systems. The time is now to give forethought to strong incentive structures to promote the safe and effective use of digital biomarkers. Generally, the verification and validation of a digital biomarker should be not construed as a one-time process, but rather, a learning digital health system should continuously collect data and handle modifications and updates overtime. Industry, researchers, regulators, clinicians, and patients have a joint responsibility to design such a learning system that can improve digital biomarker products, empower patients, and improve health and healthcare delivery for everyone

## References

[1] 114th Congress. *H.R.34—21st Century Cures Act* (2015–2016). https://www.nejm.org/doi/full/10.1056/NEJMp1615745.

[2] Byrom B (2018). Selection of and evidentiary considerations for wearable devices and their measurements for use in regulatory decision making: recommendations from the ePRO Consortium. Value Health.

[3] Zhan A (2018). Using smartphones and machine learning to quantify Parkinson disease severity: the mobile Parkinson disease score. JAMA Neurol..

[4] Kovatchev B (2018). The artificial pancreas in 2017: the year of transition from research to clinical practice. Nat. Rev. Endocrinol..

[5] Atreja, A. et al. *Mobilizing mHealth Innovation for Real-World Evidence Generation* (Duke Margolis Center for Health Policy, https://healthpolicy.duke.edu/sites/default/files/atoms/files/duke-margolis_mhealth_action_plan.pdf, 2018).

[6] Izmailova ES, Wagner JA, Perakslis ED (2018). Wearable devices in clinical trials: hype and hypothesis. Clin. Pharmacol. Ther..

[7] Parmar, A. *FDA Clears New Dexcom CGM that Requires No Patient Calibration Earlier than Expected*. https://medcitynews.com/2018/03/fda-clears-new-dexcom-cgm-requires-no-patient-calibration-earlier-expected/ (2018).

[8] Shuren J, Patel B, Gottlieb S (2018). FDA regulation of mobile medical apps. JAMA.

[9] U.S. Food and Drug Administration. *ECG App: Electrocardiograph Software for Over-the-Counter Use*. https://www.accessdata.fda.gov/cdrh_docs/pdf18/DEN180044.pdf (2018).

[10] U.S. Food and Drug Administration. *Irregular Rhythm Notification Feature: Photoplethysmograph Analysis Software for Over-the-Counter Use.* https://www.accessdata.fda.gov/cdrh_docs/pdf18/DEN180042.pdf (2018).

[11] Apple. A *Message to Our Customers about iPhone Batteries and Performance.* https://www.apple.com/iphone-battery-and-performance/ (2017).

[12] Steinhubl SR, McGovern P, Dylan J, Topol EJ (2017). The digitised clinical trial. Lancet.

[13] Martinez-Martin N, Insel TR, Dagum P, Greely HT, Cho MK (2018). Data mining for health: staking out the ethical territory of digital phenotyping. npj Digit. Med..

[14] Clinical Trials Transformative Initiative. *CTTI Unveils Recommendations for Using Mobile Technologies in Clinical Research*. https://www.ctti-clinicaltrials.org/news/ctti-unveils-recommendations-using-mobile-technologies-clinical-research (2018).

[15] Sage Bionetworks. *Elements of Informed Consent.* http://sagebionetworks.org/in-the-news/elements-informed-consent/ (2018).

[16] Torous J, Onnela JP, Keshavan M (2017). New dimensions and new tools to realize the potential of RDoC: digital phenotyping via smartphones and connected devices. Transl. Psychiatry.

[17] U.S. Food and Drug Administration. *Medical Device Safety Action Plan: Protecting Patients, Promoting Public Health*. https://www.fda.gov/downloads/AboutFDA/CentersOffices/OfficeofMedicalProductsandTobacco/CDRH/CDRHReports/UCM604690.pdf. Accessed 25 Feb 2019.

[18] Gold M (2018). Digital technologies as biomarkers, clinical outcomes assessment, and recruitment tools in Alzheimer's disease clinical trials. Alzheimers Dement..

[19] Ritchie K (2017). The midlife cognitive profiles of adults at high risk of late-onset Alzheimer's disease: the PREVENT study. Alzheimers Dement..

[20] Dowling AV, Favre J, Andriacchi TP (2012). Inertial sensor-based feedback can reduce key risk metrics for anterior cruciate ligament injury during jump landings. Am. J. Sports Med..

[21] Varela Casal, P. et al. Clinical validation of eye vergence as an objective marker for diagnosis of ADHD in children. *J. Atten. Disord.*10.1177/1087054717749931 (2018). 10.1177/1087054717749931 29357741

[22] Rajpurkar, P., Hannun, A., Masoumeh, H., Bourn, C. & Ng, A. Cardiologist-level arrhythmia detection with convolutional neural networks. arXiv preprint arXiv:1707.01836, https://arxiv.org/pdf/1707.01836.pdf (2017).

[23] Gosh SS, Ciccarelli G (2016). Speaking one's mind: vocal biomarkers of depression and Parkinson disease. J. Acoust. Soc. Am..

[24] RespApp. *Diagnosing Respiratory Disease in Children Using Cough Sounds 2 (SMARTCOUGH-C-2)*. https://www.clinicaltrials.gov/ct2/show/NCT03392363 (2018).

[25] Sage Bionetworks. *Sage Bionetworks in Collaboration with The Michael J. Fox Foundation Announce Winners in the DREAM Parkinson’s Disease Digital Biomarker Challenge*. https://www.businesswire.com/news/home/20180117006187/en (2018).

[26] Barrett MA (2017). Effect of a mobile health, sensor-driven asthma management platform on asthma control. Ann. Allergy Asthma Immunol..

[27] Wolz R, Munro J, Guerrero R, Hill DL, Dauvilliers Y (2017). Predicting sleep/wake patterns from 3-axis accelerometry using deep learning. Alzheimer Dement..

[28] Moreau A (2018). Detection of nocturnal scratching movements in patients with atopic dermatitis using accelerometers and recurrent neural networks. IEEE J. Biomed. Health Inform..

[29] Mindstrong Health. *Mindstrong Health and Takeda Partner to Explore Development of Digital Biomarkers for Mental Health Conditions*. https://www.prnewswire.com/news-releases/mindstrong-health-and-takeda-partner-to-explore-development-of-digital-biomarkers-for-mental-health-conditions-300604553.html (2018).

[30] physIQ. *physIQ.* http://www.physiq.com/resources/.

[31] Bosl WJ, Tager-Flusberg H, Nelson CA (2018). EEG analytics for early detection of autism spectrum disorder: a data-driven approach. Sci. Rep..

[32] Halcox JPJ (2017). Assessment of remote heart rhythm sampling using the AliveCor Heart Monitor to screen for atrial fibrillation: The REHEARSE-AF Study. Circulation.

[33] Kessing, L. V. *Effects of Erythropoietin on Cognition and Neural Activity in Bipolar Disorder (PRETEC-EPO)*. https://clinicaltrials.gov/ct2/show/NCT03315897 (2017).

[34] Padwal RS (2017). Validation of the Omron HEM-9210T by the ANSI/AAMI/ISO 81060-2 with two novel cuffs: wide range and extra-large. Blood Press Monit..

