# Author Correction: Developing and adopting safe and effective digital biomarkers to improve patient outcomes

**DOI:** 10.1038/s41746-019-0119-8

**Published:** 2019-05-10

**Authors:** Andrea Coravos, Sean Khozin, Kenneth D. Mandl

**Affiliations:** 1 Computational Health Informatics Program, Boston Children’s Hospital, Boston, MA USA; 2 Harvard-MIT Center for Regulatory Science, Boston, MA USA; 3 Food and Drug Administration, Silver Spring, MD USA; 4 Department of Biomedical Informatics, Harvard Medical School, Boston, MA USA

**Keywords:** Diagnostic markers, Policy

**Correction to:** *npj Digital Medicine* 10.1038/s41746-019-0090-4, published online 11 March 2019

The original version of the published Article mistakenly omitted the second affiliation for the first Author, Andrea Coravos. The first Author’s affiliation has been updated to include Harvard-MIT Center for Regulatory Science, Boston, MA, USA. This has been corrected in the HTML and PDF version of the Article.

